# Following Old Masters

**Published:** 1886-03

**Authors:** 


### FOLLOWING OLD MASTERS.

In his address before the Albany Medical College, Dr. Franklin Townsend Jr., gives the following, among the many excellent points in the course of his remarks. We cannot see, exactly why, or when he has left any point for demanding that the one medical school should be the only one, that is to give the world knowledge. We must take all, if we would join in wisdom.

At the time, however, when D'Alembert wrote, the art of medicine was enveloped in mystery and empiricism, beside fraud and imposture. "Until a comparatively recent period the doctrines and precepts of Hippocrates were followed reverentially, and the most degraded attention was paid to authority and established routine."

This folly has been properly castigated by Moliere, in his "L' Amour Medecine," in the dialogue maintained between the physician Tomes and the maid Lisette:

"*M. Thomes*—'How is the coachman?'

"*Lisette*—'Very well; he is dead.'

"*Thomes*—'Dead?'

"*Lisette*—'Yes.'

"*M. Thomes*—'That is impossible.'

"*Lisette*—'It may be impossible, but it is so.'

"*M. Thomes*—'He cannot be dead, I say.'

"*Lisette*—'I tell you he is dead and buried.'

"*M. Thomes*—'You are mistaken.'

"*Lisette*—'I saw it.'

"*M. Thomes*—'It is impossible. Hippocrates says that such diseases do not terminate till the fourteenth or twenty-first day and it is only six day since he was taken sick.'

"*Lisette*—'Hippocrates may say what he pleases, but the coachman is dead.'"

---

**In Regard to the Use of Cocoaine**, the *Kansas City Medical Record* says: "Duty compels us to call a halt on its indiscriminate use, either by hypodermic injection or by internal administration. It is going to be a worse habit to control when formed than either opium, chloral, or alcohol.

Again, it is a most powerful heart-depressant, and will, we believe, in large doses, be a powerful narcotic poison. We submit, therefore, that the profession use it with more care and judgment. Its proper sphere being probably local anæsthesia, with benefit internally, and by hypodermic injection in but a limited number of cases.

